# Association of Serum Uric Acid Concentration with Diabetic Retinopathy and Albuminuria in Taiwanese Patients with Type 2 Diabetes Mellitus

**DOI:** 10.3390/ijms17081248

**Published:** 2016-08-02

**Authors:** Ching-Chao Liang, Pi-Chen Lin, Mei-Yueh Lee, Szu-Chia Chen, Shyi-Jang Shin, Pi-Jung Hsiao, Kun-Der Lin, Wei-Hao Hsu

**Affiliations:** 1 Department of Laboratory Technology, Kaohsiung Municipal CiJin Hospital, Kaohsiung 805, Taiwan; k670806@yahoo.com.tw; 2 Division of Endocrinology and Metabolism, Department of Internal Medicine, Kaohsiung Medical University Hospital, Kaohsiung 807, Taiwan; pichli@kmu.edu.tw (P.-C.L.); lovellelee@hotmail.com (M.-Y.L.); sjshin@kmu.edu.tw (S.-J.S.); pjhsiao@cc.kmu.edu.tw (P.-J.H.); berg.kmu@gmail.com (K.-D.L.); 3 Department of Internal Medicine, Kaohsiung Municipal Hsiao-Kang Hospital, Kaohsiung 812, Taiwan; scarchenone@yahoo.com.tw; 4 Graduate Institute of Clinical Medicine, College of Medicine, Kaohsiung Medical University, Kaohsiung 807, Taiwan; 5 Division of Nephrology, Department of Internal Medicine, Kaohsiung Medical University Hospital, Kaohsiung Medical University, Kaohsiung 807, Taiwan; 6 Center for Lipid and Glycomedicine Research, Kaohsiung Medical University, Kaohsiung 807, Taiwan; 7 Department of Internal Medicine, Kaohsiung Municipal Ta-Tung Hospital, Kaohsiung 801, Taiwan

**Keywords:** diabetes mellitus, diabetic retinopathy, uric acid, albuminuria, diabetic nephropathy

## Abstract

Patients with type 2 diabetes mellitus (DM) may experience chronic microvascular complications such as diabetic retinopathy (DR) and diabetic nephropathy (DN) during their lifetime. In clinical studies, serum uric acid concentration has been found to be associated with DR and DN. The goal of this study was to evaluate the relationship between the increases in serum uric acid level and the severity of DR and albuminuria in Taiwanese patients with type 2 DM. We recorded serum uric acid concentration, the severity of DR, and the severity of albuminuria by calculating urinary albumin-to-creatinine ratio (UACR) in 385 patients with type 2 DM. In multivariate logistic regression analysis, a high uric acid concentration was a risk factor for albuminuria (odds ratio (OR), 1.227; 95% confidence interval (CI) = 1.015–1.482; *p* = 0.034) and DR (OR, 1.264; 95% CI = 1.084–1.473; *p* = 0.003). We also demonstrated that there was a higher concentration of serum uric acid in the patients with more severe albuminuria and DR. In conclusion, an increased serum uric acid level was significantly correlated with the severity of albuminuria and DR in Taiwanese patients with type 2 DM.

## 1. Introduction

Diabetic retinopathy (DR) and diabetic nephropathy (DN) are two of the chronic microvascular complications in type 2 diabetes mellitus (DM). DR is a major cause of vision loss in adults [1], causing severe morbidity in patients with diabetes, resulting in public health and economic burdens. Prolonged exposure to the metabolic changes related to diabetes may damage the microvasculature of the retina, resulting in DR [2]. DN is a major cause of end stage renal disease in many countries [3,4], and it is a life-threatening condition. Identifying a clinical marker to detect the development and progression of diabetic microvascular complications is very important to allow for early management. A correlation between serum uric acid (SUA) level and the severity of DR has been reported in patients with type 2 diabetes [5]. SUA concentration has also been reported to be associated with DN and subclinical atherosclerosis [6,7]. In addition, DR and DN have been shown to be associated with SUA concentration. However, the association between DR and DN, and SUA level has yet to be investigated in Taiwanese patients with diabetes. An elevated uric acid level is a known major risk factor of diabetic microvascular diseases. Therefore, we performed this cross-sectional study to investigate the relationships between an increase in serum uric acid level, and the severity of DR and albuminuria in Taiwanese patients with type 2 DM.

## 2. Results

Of the 385 patients included, 292 had a SUA level <7 mg/dL (low UA group), and 93 had a SUA level ≥7 mg/dL (high UA group). The clinical characteristics of the patients in the two groups are summarized in Table 1. The mean age of the high UA group was older than that in the low UA group (*p* = 0.013). The patients in the high UA group had higher systolic blood pressure (SBP), waist circumference (WC), and waist-to-hip circumference ratio (W-to-H) ratio than the patients in the low UA group (*p* = 0.015, <0.001, and 0.003, respectively). No significant differences were found between the two groups in term of coronary artery disease (CAD), cerebrovascular disease (CVD), duration of DM, diastolic blood pressure (DBP), hip circumference (HC), and body mass index (BMI). With regards to laboratory parameters, SUA, triglyceride, high-density lipoprotein (HDL) cholesterol, fasting plasma glucose, and estimated glomerular filtration rate (eGFR) were higher in the high UA group than in the low UA group (*p* < 0.001, 0.018, 0.001, 0.027, and <0.001, respectively). There were no significant differences in serum total cholesterol, low-density lipoprotein (LDL) cholesterol, and glycated hemoglobin (HbA1c) between the two groups.

In univariate logistic regression analysis, we identified that the risk of albuminuria (urinary albumin-to-creatinine ratio (UACR) ≥30 mg/gm) (Table 2) was associated with high SBP (odds ratio (OR), 1.003; 95% confidence interval (CI) = 1.019–1.047; *p* < 0.001), high W-to-H ratio (OR, 2.154; 95% CI = 1.125–4.124; *p* = 0.021), high uric acid level (OR, 1.309; 95% CI = 1.156–1.483; *p* < 0.001), high HbA1c (OR, 1.129; 95% CI = 1.012–1.258; *p* = 0.029), and low eGFR (OR, 0.980; 95% CI = 0.973–0.987; *p* < 0.001), and that the risk of DR (Table 3) was associated with a long log duration of DM (OR, 5.295; 95% CI = 2.145–13.070; *p* < 0.001), high uric acid level (OR, 1.238; 95% CI = 1.086–1.411; *p* = 0.001), high fasting plasma glucose level (OR, 1.005; 95% CI = 1.001–1.008; *p* = 0.007), high HbA1c (OR, 1.172; 95% CI = 1.045–1.315; *p* = 0.007), and low eGFR (OR, 0.992; 95% CI = 0.984–0.999; *p* = 0.026). After multivariate adjustments, the risk factors for albuminuria were high SBP (OR, 1.023; 95% CI = 1.005–1.042; *p* = 0.015), high uric acid level (OR, 1.227; 95% CI = 1.015–1.482; *p* = 0.034), high HbA1c (OR, 1.183; 95% CI = 1.010–1.385; *p* = 0.037), and low eGFR (OR, 0.984; 95% CI = 0.972–0.997; *p* = 0.014), and the risk factors for DR were a long log duration of DM (OR, 6.133; 95% CI = 2.231–16.860; *p* < 0.001), and a high uric acid level (OR, 1.217; 95% CI = 1.013–1.461; *p* = 0.035).

**Figure 1:**
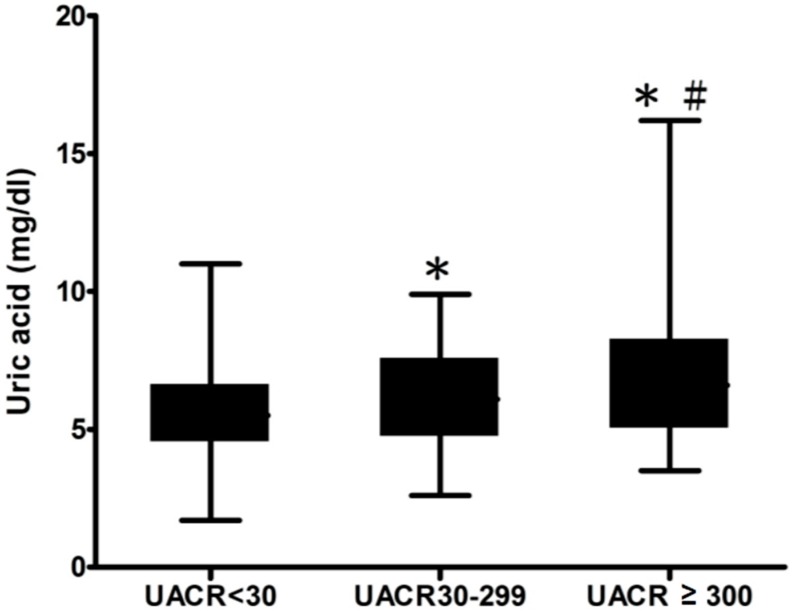
Serum uric acid (SUA) concentrations in urinary albumin-to-creatinine ratio (UACR) < 30, UACR 30–299, and UACR ≥ 300 groups. UACR < 30 group, *n* = 232, SUA = 5.6 ± 1.6 mg/dL; UACR 30–299 group, *n* = 100, SUA = 6.1 ± 1.7 mg/dL; UACR ≥ 300 group, *n* = 53, SUA = 6.9 ± 2.3 mg/dL. * *p* < 0.001, UACR 30–299 group versus UACR < 30 group; UACR ≥ 300 group versus UACR 30–299 group. ^#^ *p* < 0.001, UACR ≥ 300 group versus UACR < 30 group.

**Figure 2:**
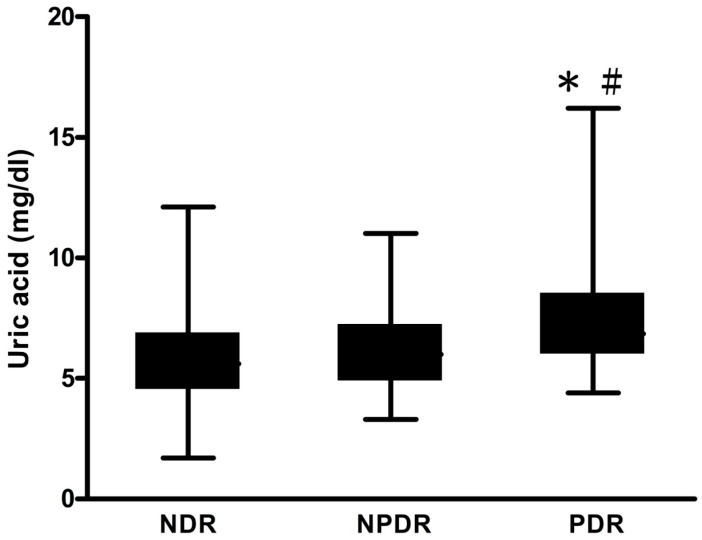
Serum uric acid (SUA) concentrations in no apparent diabetic retinopathy (NDR), non-proliferative DR (NPDR), and proliferative DR (PDR) groups. NDR group, *n* = 292, SUA = 5.7 ± 1.7 mg/dL; NPDR group, *n* = 73, SUA = 6.22 ± 1.8 mg/dL, PDR group, *n* = 20, SUA = 7.4 ± 2.5 mg/dL. * *p* < 0.001, PDR group versus NPDR group. ^#^ *p* < 0.001, PDR group versus NDR group.

We then classified all of the patients into three groups: normalbuminuria (UACR < 30 mg/gm), microalbuminuria (UACR 30–299 mg/gm), and macroalbuminuria (UACR ≥ 300 mg/gm) according to urinary albumin excretion rate (Figure 1). The level of SUA was significantly higher in the macroalbuminuria group, compared with the normalbuminuria (6.9 ± 2.3 versus 5.6 ± 1.6, *p* < 0.001), and microalbuminuria (6.9 ± 2.3 versus 6.1 ± 1.7, *p* < 0.001) groups. In the microalbuminuria group, the level of SUA was significantly higher than that in the normalbuminuria group (6.1 ± 1.7 versus 5.6 ± 1.6, *p* < 0.001). We then divided all of the patients into three groups: no apparent DR (NDR), non-proliferative DR (NPDR), and proliferative DR (PDR) according to the severity of the DR (Figure 2). The level of SUA was significantly higher in the PDR group, compared with the NDR (7.4 ± 2.5 versus 5.7 ± 1.7, *p* < 0.001), and PDR (7.4 ± 2.5 versus 6.2 ± 1.8, *p* < 0.001) groups.

## 3. Discussion

In this cross-sectional study, SUA was shown to be a risk factor for both albuminuria and DR in both univariate and multivariate logistic regression analyses. In addition, there was a trend that the more severe albuminuria or DR, the more elevated the level of SUA. These results are compatible with previous reports [5,6]. Our findings revealed that an increased level of SUA was associated with the severity of albuminuria and DR in Taiwanese patients with type 2 DM, and suggested that an elevated SUA level may reflect the severity of microvascular complications in patients with diabetes.

SUA has been reported to be elevated with increasing in the severity of DR in patients with type 2 DM [5,8]. We also found a more elevated level of SUA in the patients with more severe DR. Krizova et al. reported that vitreous concentrations of uric acid were significantly higher in patients with diabetes than in nondiabetic controls [9,10]. An increased concentration in vascular endothelial vascular factor (VEGF) in the vitreous fluid has also been demonstrated in patients with DR [11]. In studies by Selim et al. and Funatsu et al., the aqueous and vitreous levels of VEGF were found to be significantly correlated with the severity of DR [12,13]. In another clinical study by Krizova et al., uric acid concentration in vitreous fluids was reported to be significantly associated with vitreous VEGF concentration in patients with DR [10], thus supporting the hypothesis that uric acid may be a contributory factor to the pathogenesis of DR.

The SUA level has also been reported to be associated with the progression of albuminuria in patients with diabetes. Fukui et al. reported a significantly positive association between SUA concentration and the degree of urinary albumin excretion after adjusting for eGFR in men with type 2 DM [6]. Similarly, we also found a statistically significant relationship between SUA concentration and the severity of albuminuria classified into three grades. Two prospective cohort studies reported an association between SUA and the development or progression of DN in patients with type 1 DM and type 2 DM, respectively [14,15]. In addition, the level of SUA was independently associated with the progression of albuminuria to macroalbuminuria early in the course of type 1 DM [14]. A prospective study from Japan demonstrated a significant association between higher baseline SUA level and the subsequent risk of DN progression in patients with type 2 DM [15].

Several studies have reported that SUA may also play a role in diabetic peripheral neuropathy (DPN), a common microvascular complication in patients with diabetes. Higher levels of SUA in diabetic patients with peripheral neuropathy have also been reported [16,17]. Recently, a systemic review and meta-analysis reported an obvious increase in SUA levels in diabetic patients with DPN compared to those without DPN, and that hyperuricemia was significantly associated with an increased risk of DPN in patients with type 2 DM [18].

Hyperuricemia may be a marker and itself possibly be responsible for microvascular damage through inhibition of endothelial nitric oxide synthetase and activation of the renin-angiotensin system [19]. In addition, uric acid may cause microvascular disease independently of hypertension, possibly due to the direct effect of uric acid on endothelial cells and vascular smooth muscle cells [20]. In one review article, an elevated uric acid level was shown to be strongly associated with hypertension, kidney disease, metabolic syndrome, and carotid and coronary artery diseases [20], demonstrating an association between uric acid and microvascular and macrovascular diseases.

A xanthine oxidase inhibitor can inhibit the activity of xanthine oxidase, the enzyme responsible for the conversion of hypoxanthine to xanthine to uric acid. Therefore, inhibition of xanthine oxidase can reduce the production of uric acid. Xanthine oxidase inhibitors include purine analogues (e.g., allopurinol), and non-purine analogues (e.g., febuxostat, and topiroxostat). In a randomized controlled trial enrolling patients with type 2 DM and nephropathy, Momeni et al. found that the 24-h urine protein level decreased after 4 months of allopurinol administration, probably due to a decreased level of serum uric acid [21]. However, in a systemic review including eight trials of patients with or without DM, or IgA nephropathy, meta-analysis of five trials showed that changes in proteinuria from baseline were similar between the allopurinol and control arms [22]. More recently, an ongoing clinical trial conducted by David Z.I. Cherney. investigated the effect of febuxostat on renal function including changes in glomerular filtration rate in adult patients with type 1 DM [23]. Another ongoing trial conducted by Sawako Kato et al. assesses the anti-albuminuric effect of topiroxostat in patients with hyperuricemia and DN [24]. However, the effect of lowering SUA level on DR has not previously been thoroughly discussed. Therefore, a randomized controlled trial focusing on the effect of uric acid-lowering agents on albuminuria and DR in patients with diabetes is needed to investigate the effect of decrease in SUA level.

There are some limitations to this study. First, the cross-sectional design may have caused interference among the data; Second, we did not consider medications that may influence the concentration of SUA; Third, the number of enrolled subjects was small, and this may have influenced the power of the statistical analyses. Larger prospective trials are needed to assess the associations between uric acid, and DR and albuminuria; Finally, we did not evaluate diabetic neuropathy, and therefore our results cannot definitely conclude that SUA is associated with the severity of microvascular complications in patients with diabetes.

## 4. Materials and Methods

### 4.1. Subjects and Study Design

This cross-sectional study included 385 patients with type 2 DM from Kaohsiung Municipal Ci-Jin Hospital, and Kaohsiung Medical University Hospital in Taiwan from 1 September 2014 to 29 February 2016. The inclusion criteria were patients attending the hospital clinic for treatment of type 2 DM, an age 18 years or older, and a duration of DM ≥1 year. The exclusion criteria were patients with type 1 DM, those who were pregnant, those treated for cancer in the last 5 years before study enrollment, those with blood disorders causing hemolysis (e.g., hemolytic anemia), kidney transplant recipients, and those with chronic glomerulonephritis. This study was conducted according to approved guidelines. The study protocol was approved by the Institutional Review Board of Kaohsiung Medical University Hospital (KMUHIRB-E(I)-20160048), and written informed consent was obtained from each participant.

### 4.2. Demographic and Clinical Data

Demographic and medical data of the patients were collected by reviewing medical records, and included age, gender, SBP, DBP, WC, HC, W-to-H ratio, and BMI. Blood pressure was obtained from the upper limbs with the patient in a seated position using an automated device with the modified oscillometric pressure sensor method. The WC was measured at the proximate midpoint between the lower margin of the last palpable rib and the top iliac crest of the patients when standing. The HC was determined by measuring around the widest portion of the buttocks in a standing position. BMI was calculated as the ratio of weight in kilograms divided by the square of height in meters. Laboratory data were measured from fasting blood samples using an autoanalyzer (Roche Diagnostics GmbH, D-68298 Mannheim COBAS Integra 400, Mannheim, Germany). Serum creatinine was measured by using the compensated Jaffé method in a Roche/Integra 400 Analyzer. The abbreviated Modification of Diet in Renal Disease (MDRD) Study Group equation was used to calculate the eGFR: eGFR (mL/min/1.73 m^2^) = 186.3 × (serum creatinine^−1.154^) × (age^−0.203^) × 0.742 if female [25]. Urine albumin and creatinine were measured from a spot urine sample using an autoanalyzer (COBAS Integra 400 plus; Roche Diagnostics, Mannheim, Germany). Albuminuria was defined as UACR of ≥30 mg/gm. The ratio stood for urinary albumin excretion rate. The definitions of normalbuminuria, microalbuminuria, and macroalbuminuria were UACR < 30 mg/gm, UACR 30–299 mg/gm, and UACR ≥ 300 mg/gm, respectively.

### 4.3. Diabetes Retinopathy

DR was evaluated by experienced ophthalmologists while the patients’ pupils were dilated. DR was classified as NDR, NPDR, and PDR. PDR was defined as the presence of the neovascularization of the retinal vessels and the complications of this neovascularization such as preretinal hemorrhage, vitreous hemorrhage, and traction retinal detachment. NPDR consisted of nerve fiber layer infarcts, intraretinal hemorrhage, hard exudates, and microvascular abnormalities such as microaneurysms in the retina without the presence of the neovascularization in the retina. Patients without these abnormalities in the retina were classified in the NDR group.

### 4.4. Statistical Analyses

Data are expressed as mean ± standard deviation (SD). The Statistical Package for Social Science software (SPSS for Windows, version 19.0, International Business Machines Corporation (IBM), Armonk, NY, USA) was used to perform all statistical analyses. The Student’s *t* test was used for continuous variables, and the χ^2^ test was used for categorical variables. Binary logistic regression analysis was used to assess the influence of continuous and categorical variables on UACR, and DR to evaluate the risk factors for DN and DR. We also tried to run the log-binomial regression. Most of the coefficients from log-binomial regression were similar to those from logistic regression. However, some coefficients were undetectable due to stop of integration by Newton’s method. Therefore, we still run the logistic regression. A one-way analysis of variance (ANOVA) test was performed to compare the mean levels of SUA in two sets of three groups (albuminuria and DR, as shown in Figure 1 and Figure 2). A *p* value less than 0.05 was considered to be statistically significant.

## 5. Conclusions

In conclusion, we found that an increased SUA level was significantly correlated with the severity of albuminuria and DR in Taiwanese patients with type 2 DM. Multivariate logistic regression analysis showed that SUA was a risk factor for both albuminuria and DR. Uric acid may play a role in the pathogenesis of diabetic microvascular diseases. Patients with type 2 diabetes mellitus often had coexisting microvascular complications when the diagnosis of diabetes mellitus was made. Therefore, identifying a clinical surrogate for the severity of diabetic microvascular complications is needed. Our findings suggested that in clinical practice, the SUA level obtained from diabetic patients may reflect the severity of the current microvascular complications in patients with type 2 DM. Regular measurements of SUA level as a potential marker for the severity of microvascular diseases may be beneficial for patients with diabetes.

## Figures and Tables

**Table 1:** Comparison of clinical characteristics between patients with serum uric acid (SUA) <7 and ≥7 mg/dL.

Characteristics	All Patients (*n* = 385)	SUA < 7 mg/dL (*n* = 292)	SUA ≥ 7 mg/dL (*n* = 93)
Age (year)	64.6 ± 12.1	63.7 ± 11.6	67.3 ± 13.3 *
Male gender (%)	49.6	43.5	68.8
CAD (%)	6.6	7.2	4.3
CVD (%)	3.8	2.7	7.2
Duration of DM (years)	9 (5–16)	9 (4–15)	11 (6–16)
Systolic BP (mmHg)	138.6 ± 17.8	137.3 ± 17.1	142.7 ± 19.6 *
Diastolic BP (mmHg)	76.1 ± 11.9	76.0 ± 11.5	76.1 ± 13.0
WC (cm)	92.6 ± 11.1	91.0 ± 10.4	97.5 ± 11.7 **
HC (cm)	100.0 ± 10.0	99.3 ± 10.2	102.4 ± 9.4
W-to-H ratio	0.93 ± 0.07	0.92 ± 0.07	0.95 ± 0.06 *
BMI (kg/m^2^)	26.4 ± 4.6	26.1 ± 4.4	27.3 ± 5.2
Laboratory parameters			
Uric acid (mg/dL)	5.9 ± 1.8	5.1 ± 1.1	8.3 ± 1.3 **
Triglyceride (mg/dL)	121 (83.5–172)	117 (78–164)	144 (99.5–206.5) *
Total cholesterol (mg/dL)	180.6 ± 47.7	179.9 ± 46.4	182.9 ± 51.6
HDL-cholesterol (mg/dL)	45.0 ± 12.9	46.4 ± 13.0	40.8 ± 11.6 *
LDL-cholesterol (mg/dL)	101.7 ± 35.4	100.8 ± 33.2	104.5 ± 41.6
Fasting glucose (mg/dL)	154.8 ± 64.3	158.4 ± 67.3	143.4 ± 52.6 *
HbA1c (g/dL)	7.6 ± 1.9	7.6 ± 1.9	7.5 ± 1.9
eGFR (mL/min/1.73 m^2^)	78.6 ± 33.0	84.5 ± 31.5	60.0 ± 30.7 **
Urinary albumin-to-creatinine ratio (UACR) (mg/gm) (*p* < 0.001)			
<30	60.3	66.4	40.9
30–300	26.0	22.9	35.5
≥300	13.8	10.6	23.7
Diabetic retinopathy (DR) (*p* = 0.051)			
NDR	75.8	78.1	68.9
NPDR	19.0	18.2	21.5
PDR	5.2	3.8	9.7

* *p* < 0.05, ** *p* < 0.001 compared to patients with SUA <7 mg/dL. CAD, coronary artery disease; CVD, cerebrovascular disease; DM, type 2 diabetes mellitus; BP, blood pressure; WC, waist circumference; HC, hip circumference; W-to-H, waist-to-hip circumference ratio; BMI, body mass index; HDL, high-density lipoprotein; LDL, low-density lipoprotein; HbA1c, glycated hemoglobin; eGFR, estimated glomerular filtration rate; NDR, no apparent DR; NPDR, non-proliferative DR.

**Table 2:** Risk factors for urinary albumin-to-creatinine ratio (UACR) ≥ 30 mg/gm using binary logistic regression analysis.

Parameters	Univariate		Multivariate	
	OR (95% CI)	*p*	OR (95% CI)	*p*
Age (per 1 year)	1.009 (0.992–1.027)	0.278	0.987 (0.956–1.019)	0.424
Male gender (versus female)	1.486 (0.986–2.240)	0.059	0.910 (0.457–1.812)	0.788
CAD	0.703 (0.259–1.907)	0.489	-	-
CVD	1.918 (0.571–6.439)	0.292	-	-
Log duration of DM (per 1 year)	1.623 (0.839–3.138)	0.150	-	-
Systolic BP (per 1 mmHg)	1.033 (1.019–1.047)	<0.001	1.023 (1.005–1.042)	0.015
Diastolic BP (per 1 mmHg)	1.013 (0.995–1.032)	0.156	-	-
WC (per 1 cm)	1.012 (0.987–1.039)	0.350	-	-
HC (per 1 cm)	0.988 (0.960–1.017)	0.404	-	-
W-to-H ratio (per 0.01)	2.154 (1.125–4.124)	0.021	1.816 (0.854–3.861)	0.121
BMI (per 1 kg/m^2^)	1.007 (0.959–1.058)	0.766	-	-
Laboratory parameters				
Uric acid (per 1 mg/dL)	1.309 (1.156–1.483)	<0.001	1.227 (1.015–1.482)	0.034
Log Triglyceride (per 1 mg/dL)	1.339 (0.614–2.921)	0.463	-	-
Total cholesterol (per 1 mg/dL)	0.999 (0.994–1.003)	0.524	-	-
HDL-cholesterol (per 1 mg/dL)	0.986 (0.969–1.004)	0.120	-	-
LDL-cholesterol (per 1 mg/dL)	0.996 (0.990–1.002)	0.178	-	-
Fasting glucose (per 1 mg/dL)	1.002 (0.998–1.005)	0.348	-	-
HbA1c (per 1%)	1.129 (1.012–1.258)	0.029	1.183 (1.010–1.385)	0.037
eGFR (per 1 mL/min/1.73 m^2^)	0.980 (0.973–0.987)	<0.001	0.984 (0.972–0.997)	0.014

Values express as odds ratios (OR) and 95% confidence interval (CI). Abbreviations are same as Table 1.

**Table 3:** Risk factors for diabetic retinopathy using binary logistic regression analysis.

Parameters	Univariate		Multivariate	
	OR (95% CI)	*p*	OR (95% CI)	*p*
Age (per 1 year)	0.992 (0.973–1.011)	0.392	0.974 (0.948–1.001)	0.060
Male gender (versus female)	0.862 (0.541–1.373)	0.532	0.920 (0.495–1.709)	0.793
CAD	0.858 (0.275–2.679)	0.792	-	-
CVD	0.315 (0.04–2.506)	0.275	-	-
Log duration of DM (per 1 year)	5.295 (2.145–13.070)	<0.001	6.133 (2.231–16.860)	<0.001
Systolic BP (per 1 mmHg)	1.014 (1.000–1.028)	0.052	-	-
Diastolic BP (per 1 mmHg)	1.005 (0.984–1.026)	0.641	-	-
WC (per 1 cm)	0.975 (0.946–1.006)	0.109	-	-
HC (per 1 cm)	0.974 (0.941–1.008)	0.127	-	-
W-to-H ratio (per 0.01)	0.952 (0.478–1.894)	0.888	-	-
BMI (per 1 kg/m^2^)	0.961 (0.906–1.019)	0.185	-	-
Laboratory parameters				
Uric acid (per 1 mg/dL)	1.238 (1.086–1.411)	0.001	1.217 (1.013–1.461)	0.035
Log Triglyceride (per 1 mg/dL)	1.550 (0.644–3.732)	0.328	-	-
Total cholesterol (per 1 mg/dL)	1.005 (1.000–1.009)	0.057	-	-
HDL-cholesterol (per 1 mg/dL)	1.007 (0.988–1.027)	0.469	-	-
LDL-cholesterol (per 1 mg/dL)	1.001 (0.995–1.008)	0.701	-	-
Fasting glucose (per 1 mg/dL)	1.005 (1.001–1.008)	0.007	1.000 (0.995–1.005)	0.966
HbA1c (per 1%)	1.172 (1.045–1.315)	0.007	1.159 (0.963–1.395)	0.118
eGFR (per 1 mL/min/1.73 m^2^)	0.992 (0.984–0.999)	0.026	0.997 (0.986–1.008)	0.605

Values express as odds ratios (OR) and 95% confidence interval (CI). Abbreviations are same as Table 1.
